# LSD1 Demethylates and Destabilizes Autophagy Protein LC3B in Ovarian Cancer

**DOI:** 10.3390/biom14111377

**Published:** 2024-10-29

**Authors:** Mingyang Li, Jie Feng, Kangrong Zhao, Ting Huang, Bowen Zhang, Yifan Yang, Aiqin Sun, Qiong Lin, Genbao Shao

**Affiliations:** 1 Institute of Urinary System Diseases, The Affiliated People’s Hospital, Jiangsu University, 8 Dianli Road, Zhenjiang 212002, China; limy@ujs.edu.cn; 2 Department of Basic Medicine, School of Medicine, Jiangsu University, Zhenjiang 212013, China

**Keywords:** demethylation, proteasome degradation, epigenetic modification, prognosis, autophagy

## Abstract

Autophagy is a complex cellular process that can either promote or inhibit cancer progression and development, depending on the context and molecular regulation involved. This study investigates how LSD1 regulates autophagy in ovarian cancer by interacting with the autophagy protein LC3B. Utilizing the bioinformatic analysis of TCGA, CPTAC, and GEO datasets, as well as immunohistochemistry in ovarian cancer patients, we explored the expression association between LSD1 and LC3B. Molecular mechanisms were further analyzed using Western blotting, immunoprecipitation, and GST pull-down assays. Our findings reveal that LSD1 binds to LC3B via its SWIRM domain, and high levels of LSD1 are closely associated with aggressive ovarian cancer and poor patient outcomes. Mechanistically, LSD1 demethylates LC3B, leading to decreased LC3B stability. The observed inverse correlation between LSD1 expression and LC3B protein levels in clinical samples underscores the need for further investigation to elucidate how reduced LC3B protein levels induced by LSD1 demethylation may contribute to ovarian cancer.

## 1. Introduction

Autophagy is a catabolic process of cellular degradation which targets cytoplasmic material such as damaged organelles, external bacteria, aggregated proteins, and others [1,2] in response to many environmental challenges, mainly oxidative stress and nutritional starvation [3,4]. It has been demonstrated that autophagy modulation serves as a double-edged sword in both tumor promotion and suppression in various types of cancers [5,6]. Understanding the complicated molecular regulation of autophagy associated with tumor development and therapy resistance is pivotal for establishing new approaches to cancer treatment. Autophagy starts with the formation of double-membrane autophagosomes, in which cytoplasmic constituents are engulfed and delivered to lysosomes for degradation [7]. MAPLC3 (microtubule-associated protein 1 light chain 3, simply LC3), a mammalian homolog of the yeast autophagy protein ATG8, is a crucial regulator of autophagy in the generation of the autophagosome membrane [8], the sequestration of different cytosolic cargoes [9], and the fusion of autophagosome and lysosome [10]. These activities are based on the functional LC3-II localized on both the outer and inner surfaces of the autophagosome [11]. The membrane-bound LC3-II, as a marker of autophagosomes, is converted by covalent modification of cytosolic LC3-I with phosphatidylethanolamine [12].

Post-translational modifications (PTMs) of autophagy-related (ATG) proteins are critical for the regulation of different aspects of autophagy [13,14]. Phosphorylation of ATG proteins is the most intensively investigated PTM in controlling autophagy levels, followed by acetylation and ubiquitination [15]. Methylation of proteins on arginine and lysine residues by methyltransferases is one of the common types of PTM, and the methyl groups can be removed by specific demethylases [16]. Various histone methylation and multiple signaling pathways governed by non-histone protein methylation function as a switch to regulate autophagy, whereas there has thus far been relatively limited research on ATG protein methylation [17,18]. Emerging evidence strongly supports that a transition between the methylation and demethylation status of ATG proteins acts as a novel regulatory mechanism of autophagy by controlling their protein functions. ATG16L methylated by the lysine methyltransferase SETD7 impairs its binding to the ATG5-ATG12 conjugate, resulting in autophagy inhibition, while the methyl marks of ATG16L can be removed by LSD1 [19]. FIP200 methylated by methyltransferase SETD2 prevents its degradation mediated by ubiquitination, maintaining autophagy activity [20]. The impacts of acetylation/deacetylation and ubiquitination of LC3 on autophagy have been reported [21,22]. However, the methylation or demethylation of LC3 and its effects remain unknown.

LSD1 is the first identified histone demethylase, which functions as an amine oxidase to specifically demethylate histone H3 lysine 4 (H3K4) through a flavin adenine dinucleotide-dependent oxidative reaction [23]. The association of LSD1 with the cofactor CoREST and histone deacetylase 1/2 stimulates its ability to mediate H3K4 demethylation, promoting transcriptional repression [24]. In contrast, LSD1, when binding to the androgen receptor (AR), acts as a transcriptional coactivator of AR target genes by demethylating H3K9 [25]. In addition to the role in histone modifications, LSD1 demethylates multiple fundamental non-histone proteins, contributing to the regulation of different cellular pathways activated in several biological processes, including tumor progression [26,27]. The target proteins demethylated by LSD1 generate diverse consequences. For instance, demethylation of p53 K370me2 by LSD1 inhibits the interaction of p53 with the cofactor p53-binding protein 1, thus repressing pro-apoptotic activity [28], whereas demethylation of HIF-1α at K391 protects HIF-1α against ubiquitin-mediated protein degradation, enhancing tumor angiogenesis [29]. The high expression of LSD1 is correlated with poor prognosis of a variety of cancer types, including prostate, breast, neuroblastoma, lung, and bladder cancers [30], playing an essential role in distinct aspects of cancer [31]. We have reported that the overexpression of LSD1 promotes ovarian cancer cell migration driven by EGF signaling [32]. Furthermore, our previous study revealed that LSD1 expression negatively regulates autophagy in ovarian cancer cells [33]. However, the molecular mechanism underlying LSD1 epigenetic regulation of ATG gene expression and proteins in ovarian cancer remains poorly understood.

In this research, for the first time we explore the regulation of the autophagy biomarker protein LC3B, a subfamily member of LC3, by lysine demethylation through LC3B-interacting proteins. We determined that LSD1, a facilitator for ovarian tumor progression, directly interacts with LC3B. Mechanistic analyses revealed that LSD1 demethylates LC3B and promotes the protein degradation of LC3B. Of note, LSD1 expression is inversely correlated with the protein level of LC3B, thereby suggesting the role of LSD1 in PTMs of LC3B. Our studies have revealed a novel PTM of the autophagy protein LC3B that is mediated by LSD1.

## 2. Materials and Methods

### 2.1. Cell Line and Culture Condition

The human ovarian epithelial carcinoma cell lines SKOV3 and HO8910 were generously gifted by Dr. Qixiang Shao at Jiangsu University (Zhenjiang, China). The HEK293T cells were obtained from BOSTER Biological Technology (Wuhan, China). These three cell lines were cultured in Dulbecco’s modified Eagle’s medium with high glucose (Hyclone, Logan, UT, USA), supplemented with 10% heat-inactivated fetal bovine serum (ExCell Bio, Shanghai, China) and 100 U/mL penicillin-streptomycin (Hyclone, Logan, UT, USA). All mycoplasma-free cell lines were cultured under standard conditions (37 °C with 5% CO_2_), maintained in the logarithmic growth phase to optimize transfection efficiency, and utilized at low passage numbers to preserve their inherent phenotypic characteristics.

### 2.2. Chemical Reagents and Antibodies

The following reagents were used: electrochemiluminescence (ECL) reagents were purchased from Beyotime (Shanghai, China). Polybrene and doxycycline (Dox), cycloheximide (CHX), dimethyl sulfoxide (DMSO), puromycin, and 4’,6-diamidino-2-phenylindole (DAPI) were purchased from Sigma-Aldrich (St. Louis, MO, USA). The LSD1 inhibitor tranylcypromine (TCP) was obtained from Biomol International (Hamburg, Germany). The antibodies used were as follows: anti-LC3B (sc-376404) and anti-My (sc-40) from Santa Cruz (Dallas, TX, USA); anti-LSD1 (2184S) and anti-HA (3724S) from Cell Signaling Technology (Danvers, MA, USA); anti-α-tubulin (BS1699) from Bioworld Technology (Bloomington, MN, USA); and anti-pan methyl Lysine (ab7315) from Abcam (Cambridge, United Kingdom).

### 2.3. Plasmid Construction and Transfection

The human *MAP1LC3B* cDNA was inserted into pcDNA-Myc and GST vector pGEX-4T-3, respectively. pCMV3-HA-LSD1 was purchased from Sino Biological (HG13721-CY, Beijing, China). Different truncated fragments of human *LSD1* cDNA were amplified by PCR and subcloned into the pCMV3-HA vector. All of the constructed plasmids were verified correct by DNA sequencing. For transfection, the cells were plated one day prior to transfection. The transfection reagent Lipofectamine 2000 (Invitrogen, Carlsbad, CA, USA) was used for all the transfection reactions, as described previously [34]. In brief, plasmid DNA (5 to 6 μg/60 mm dish) was premixed with Lipofectamine 2000 and incubated at room temperature (RT) for 5 min. The cells were incubated with the mixture of plasmid DNA and the transfection reagent for 2 days, followed by cell lysis for protein preparation.

### 2.4. Virus Packaging and Transduction

The virus packaging was performed as described previously [35]. Briefly, HEK293T cells were cotransfected with lentiviral plasmids, pHR’-CMV-8.2ΔVPR, and pHR’-CMV-VSVG lentiviral packaging plasmids using the Lipofectamine 2000 transfection reagent. The culture medium containing lentivirus was harvested every 24 h for three times post-transfection, then clarified by centrifugation at 1000× *g* for 5 min, and added to infect target cells with 6 µg/mL polybrene.

### 2.5. Creation of Dox-Inducible LSD1 Knockdown and Overexpression Cell Lines

The inducible *LSD1* knockdown cell lines of SKOV3 and HO8910 were generated as previously described [35,36]. Briefly, the constructed plasmid pLKO-Tet-On-shLSD1 was packaged into lentiviral particles. After infection twice, cells were selected with 2 µg/mL puromycin for 3 days and then maintained with 1 µg/mL puromycin for one week until stable clones were created.

### 2.6. Immunoprecipitation and Western Blotting

For the extraction of total lysates of cellular proteins, the cells were agitated using Mammalian lysis buffer (40 mM HEPES (pH 7.4), 100 mM NaCl, 1% Triton X-100, 25 mM glycerol phosphate, 1 mM sodium orthovanadate, 1 mM EDTA) supplemented with protease inhibitors (10 μg/mL aprotinin, and 10 μg/mL leupeptin) for 30 min on ice, and the cell debris was removed by centrifugation at 12,000 rpm for 15 min at 4 °C. For immunoprecipitation, the lysates were incubated with primary antibodies against targeting proteins with rotation for 30 min, followed by the addition of protein G/protein A agarose (Millipore, Burlington, MA, USA) and incubation with rotation for 3 h at 4 °C. The immunocomplexes were washed 3 times in lysis buffer. The immunoprecipitated proteins or the cell lysates were denatured with SDS-PAGE protein loading buffer by boiling for 8 min. All protein samples were separated by 8–12% SDS-PAGE, transferred to PVDF membranes of 0.2/0.45-µm pore size (Millipore, Burlington, MA, USA). The immunoblot was performed as described previously [33,36]. Briefly, the membranes were blocked in 5% non-fat dry milk/TBST for 1 h at RT. After hybridization with primary antibodies overnight at 4 °C and the corresponding secondary antibodies for 1 h at RT, the images were visualized through chemiluminescence by ECL reagents. The band intensity of the Western blot was quantified using ImageJ software (version 1.54j, National Institutes of Health, Bethesda, MD, USA) and normalized to the loading control of α-tubulin. The sample from the Dox-untreated group at 0 hours of CHX treatment was set as the reference, standardized to a value of 1.00.

### 2.7. Purification of Recombinant Protein and GST Affinity Precipitation Assay

GST fusion proteins were induced to express in *E. coli* DH5α cells by isopropyl-β-d-thiogalactopyranoside (IPTG). The bacterial culture was centrifuged and then resuspended in bacterial lysis buffer (20 mM Tris-HCl, pH8.0, 0.5% Triton X-100, 100 mM NaCl, 10 mM MgCl_2_, 250 μg/mL lysozyme, 10 μg/mL leupeptin and aprotinin). Vigorous sonication was performed, followed by centrifugation at 12,000 rpm for 15 min at 4 °C. The cleared supernatant was incubated with glutathione-agarose beads with rotation for 3 h at 4 °C. The beads binding GST fusion proteins were washed in bacterial lysis buffer three times. The expression quality of beads-purified GST fusion proteins was verified by Coomassie blue staining. These beads were then mixed with mammalian cell lysates by rotation for 3 h at 4 °C, washed in mammalian lysis buffer three times, and resuspended with SDS-PAGE protein loading buffer. The precipitated proteins were analyzed by SDS-PAGE and Western blotting.

### 2.8. Immunofluorescence

Cells were grown on round cover glasses placed in a 24-well plate after transfection with the indicated plasmids. The cells were washed with PBS, fixed with 4% paraformaldehyde, permeabilized with 0.5% Triton X-100, and blocked in 3% BSA in PBS. Cells were incubated with indicated primary antibodies at 4 °C overnight and fluorescence-conjugated secondary antibodies for 2 h at RT. After the staining of the nuclei with DAPI, images were acquired using a Nikon inverted fluorescent microscope (Nikon Instruments Inc., Melville, NY, USA).

### 2.9. Tissue Specimens and Immunohistochemistry (IHC)

Tissue microarrays including a total of 96 cases of ovarian tumor and normal tissues were used for the detection of LSD1 and LC3B by IHC staining. The tissue microarray chips utilized in this study were procured from the Zhuoli Biotechnology Company (Shanghai, China). The supplier ensured that informed consent was rigorously obtained from all participating subjects prior to sample collection. The standard procedure was performed to determine the expression levels of LSD1 and LC3B in the ovarian tissue samples. In brief, formaldehyde-fixed and paraffin-embedded tissue microarray sections were first placed in a 60 °C oven for 2 h, followed by deparaffinization and rehydration in xylene and ethanol wash bath. Antigen retrieval was achieved by covering the slides fully with the boiling 10 mM sodium citrate buffer pH 6.0 for 15 min inside one microwave oven. Then, the slides were allowed to cool slowly to RT. Endogenous peroxidases were quenched by immersing the sections with enough drops of freshly prepared 3% hydrogen peroxide in methanol for 10 min. After being blocked in normal goat serum for 20 min, the sections were coated with primary antibody overnight at 4 °C, followed by incubation with enzyme-conjugated secondary antibody for 30 min at RT. DAB was applied to develop the staining signal as a chromogen until the desired color reaction was observed under a microscope, and then hematoxylin was added for counterstaining. The IHC staining images in the specimens were obtained at ×400 magnification by the NanoZoomer S210 digital slide scanner (Hamamatsu Photonics, Hamamatsu, Japan). The percentage of LSD1 or LC3B-positive cells and the staining intensity were analyzed, and the IHC staining scores were produced by Visiopharm software (version 2023.01, Visiopharm A/S, Hoersholm, Denmark).

### 2.10. Analysis of TCGA and GEO Datasets

The microarray datasets of TCGA_OV (Ovarian Cancer Cohort), Etemadmoghadam [37], GSE26712, GSE18520, GSE27651, and GSE54388, retrieved from TCGA and GEO, were used to analyze *LSD1* mRNA expression levels associated with clinical pathological characteristics in ovarian cancers. The relationship between LSD1 and LC3B protein expression levels was evaluated using protein expression data from the Clinical Proteomic Tumor Analysis Consortium (CPTAC) data portal. The mRNA and protein levels between the two groups were compared using *t*-tests. The following five ovarian cancer datasets were selected for prognostic analyses: TCGA_OV, GSE9891, GSE26193, GSE63885, and GSE18520. The analysis of overall survival (OS) rate between the ovarian cancer patients stratified by LSD1 high and low expression was performed using the Kaplan–Meier (KM) method with GraphPad Prism 10 (Dotmatics, Boston, MA, USA). The cut-off value of the LSD1 expression levels was determined based on the highest significance, which is the lowest false discovery rate (FDR) using the Benjamini–Hochberg method. The Mantel–Cox log-rank test was used to examined the significance of differences between survival curves. The factors (including LSD1 expression levels) correlated with OS outcomes were investigated using univariate and multivariate Cox proportional hazards models with IBM SPSS Statistics 29 (IBM, Armonk, NY, USA).

### 2.11. Statistical Analysis

Univariate and multivariate analyses were performed with IBM SPSS Statistics 29 software, and the remaining data were analyzed using the GraphPad Prism 10 software. The data values are presented as box-and-whisker plots, showing the median, interquartile range, and the minimum and maximum values. The differences between two groups were statistically analyzed by the Mann–Whitney test (two-tailed), and those between more than two groups were analyzed by the Kruskal–Wallis test. A significant difference in staining percentages between LSD1 and LC3B was determined using the Wilcoxon matched-pairs signed-rank test. * *p* < 0.05 was considered as a statistically significant difference plus ** *p* < 0.01, *** *p* < 0.001 and **** *p* < 0.0001.

## 3. Results

### 3.1. LSD1 Binds with LC3B via Its SWIRM Domain

**Figure 1:**
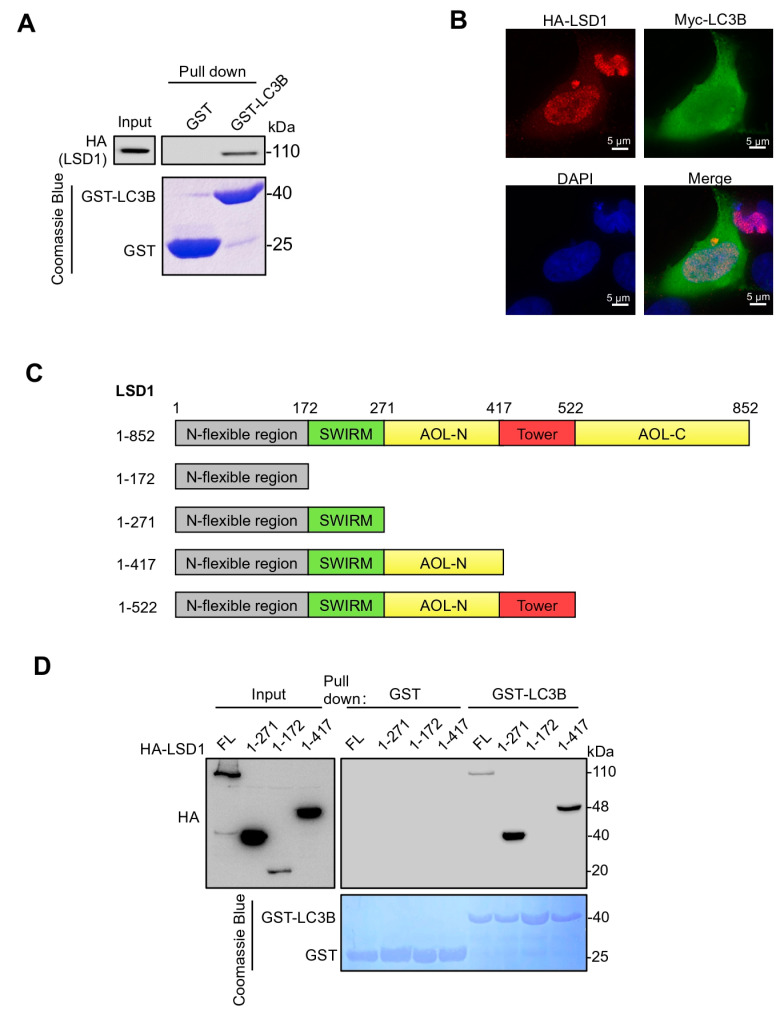
LSD1 interacts with LC3B through its SWIRM domain. (**A**) Bacterially produced GST-LC3B was used to pull down HA-LSD protein expressed in HEK293T cells. (**B**) Immunofluorescence staining analysis of LSD1 (red) and LC3B (green) in HEK293T cells following co-transfection with expression vectors for HA-LSD1 and Myc-LC3B. DAPI was used to label the nuclei. Scale bar, 5 μm. (**C**) Schematic illustration of N-terminal HA-tagged full-length (FL) LSD1 and its truncation constructs (ranges indicate chain of amino acids present in construct). The numbers labeled in LSD1 structure sketches indicate the amino acid residue positions. (**D**) The FL or various truncation mutants of HA-LSD1 shown in (**C**) were incubated with GST-LC3B. The immunoprecipitation and Western blotting analyses were carried out to detect interaction with anti-HA antibody. Original Western blot images are available in Supplementary Materials.

Preliminary pull-down experiments detected an interaction between LSD1 and LC3B, but not with LC3A or LC3C. To further validate this finding, the interaction between LC3B and LSD1 was detected by GST-pull down in the HEK293T cell line with stable HA-LSD1 overexpression (Figure 1A). The immunofluorescence result showed that LSD1 was co-localized with LC3B, and LSD1 was distributed pre-dominantly in the nucleus whereas the majority of LC3B was located in the cytoplasm (Figure 1B). To identify which domain of LSD1 directly binds to LC3B, we expressed the full-length and several fragments of HA-LSD1 (Figure 1C) and then performed a GST-LC3B affinity precipitation assay. All truncation fragments of HA-LSD1 were successfully precipitated with GST-LC3B, with the exception of the fragment encompassing amino acids 1-172. This observation suggests that the SWIRM domain of LSD1, which includes amino acids 172-271, is responsible for binding to LC3B (Figure 1D).

### 3.2. High-Level LSD1 Is Closely Associated with Aggressive Ovarian Cancer

**Figure 2:**
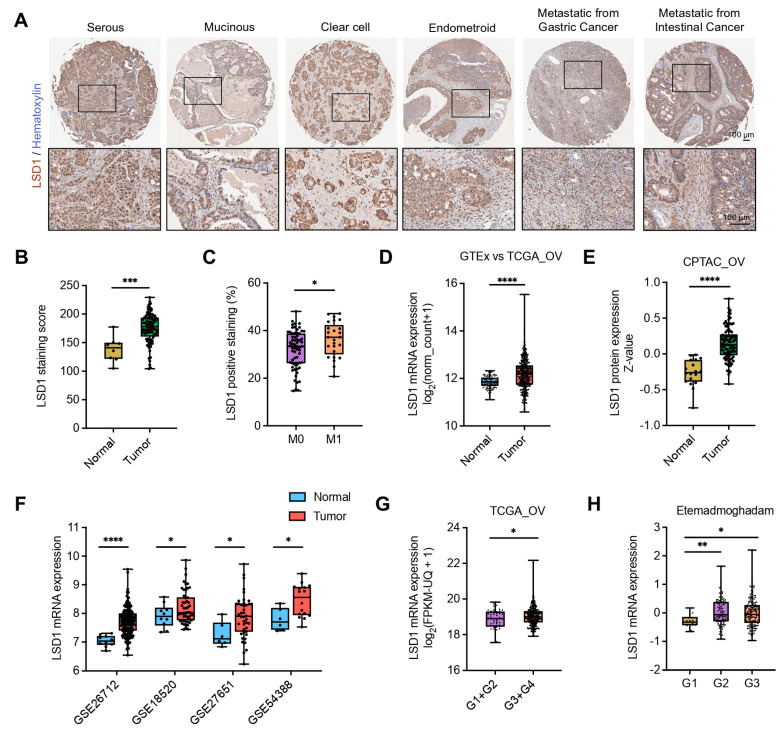
LSD1 is highly and aggressively expressed in ovarian tumors of different types. (**A**) Representative IHC images for LSD1 staining in different histotypes of ovarian cancers. Scale bar, 100 μm. The rectangle boxes shown in each IHC image in the upper panel indicate the areas that have been magnified in the lower panel for clearer visualization of the antibody staining. (**B**) Quantification of LSD1 staining score of normal ovary and ovarian tumors in IHC assay of tissue microarray. (**C**) Quantification of percentage of LSD1-positive staining of M0 and M1 metastasis stage in IHC assay of ovarian tissue microarray. (**D–F**) The comparison of *LSD1* mRNA and protein expression between ovarian cancer tissues and normal controls in TCGA ovarian cancer cohort (TCGA_OV) (**D**), a series of GEO datasets (**F**), and CPTAC protein data (**E**). (**G**,**H**) mRNA expression levels of *LSD1* at different stages of ovarian cancers of different datasets: TCGA_OV (**G**) and the Etemadmoghadam-studied dataset (**H**). * represents *p* < 0.05, ** represents *p* < 0.01, *** represents *p* < 0.001, and **** represents *p* < 0.0001.

To determine the role of LSD1 in the pathogenesis of ovarian cancer, we performed IHC staining on the ovarian cancer tissue array comprising 96 cases (including normal controls). We found that various histologic types of epithelial ovarian cancer, including serous, mucinous, clear cell, endometrioid, ovarian metastasis from gastric cancer, and intestinal cancer, showed strong LSD1 expression in IHC staining samples (Figure 2A). LSD1 is significantly upregulated in ovarian tumors when comparing LSD1-positive cases between ovarian cancer patients and healthy individuals (Supplementary Table S1). Further statistical analysis revealed a strong correlation between LSD1 overexpression and the pathological characteristics of ovarian tumors, including patient age, FIGO stage, and M category (Supplementary Table S2). These findings strongly suggest that LSD1 plays a crucial role in the progression and metastasis of ovarian cancer. Subsequently, we examined the LSD1 staining scores in the IHC samples, and the findings corroborate this conclusion. IHC analysis revealed that ovarian cancer tissues exhibited significantly higher LSD1 expression compared to normal ovarian tissues (Figure 2B). Additionally, LSD1 expression was markedly elevated in the M1 stage compared to the M0 stage of ovarian cancer metastasis (Figure 2C), indicating a correlation between LSD1 expression and ovarian cancer malignancy. Next, we explored the bioinformatics data from ovarian cancer cases that were publicly available online. The *LSD1* mRNA expression level was significantly higher in TCGA ovarian cancer than in GTEx normal ovarian tissue (Figure 2D). This finding was consistent with the results of the mRNA expression analysis of selected GSE datasets (GSE26712, GSE18520, GSE27651, and GSE54388) from GEO, which contained RNA microarray data from ovarian cancer patients and normal controls (Figure 2F). The analysis of the CPTAC dataset showed that in the ovarian tumor group, LSD1 protein expression was significantly higher than in the normal group (Figure 2E). Further analysis of RNA-related datasets revealed a positive correlation between the mRNA expression of *LSD1* and the tumor grade or FIGO stage of ovarian cancer (Figure 2G,H and Figure S1). Collectively, our data show that the LSD1 expression is frequently upregulated in ovarian cancer.

### 3.3. High LSD1 Expression Is Associated with Poor Outcomes of Patients with Ovarian Cancer

**Figure 3:**
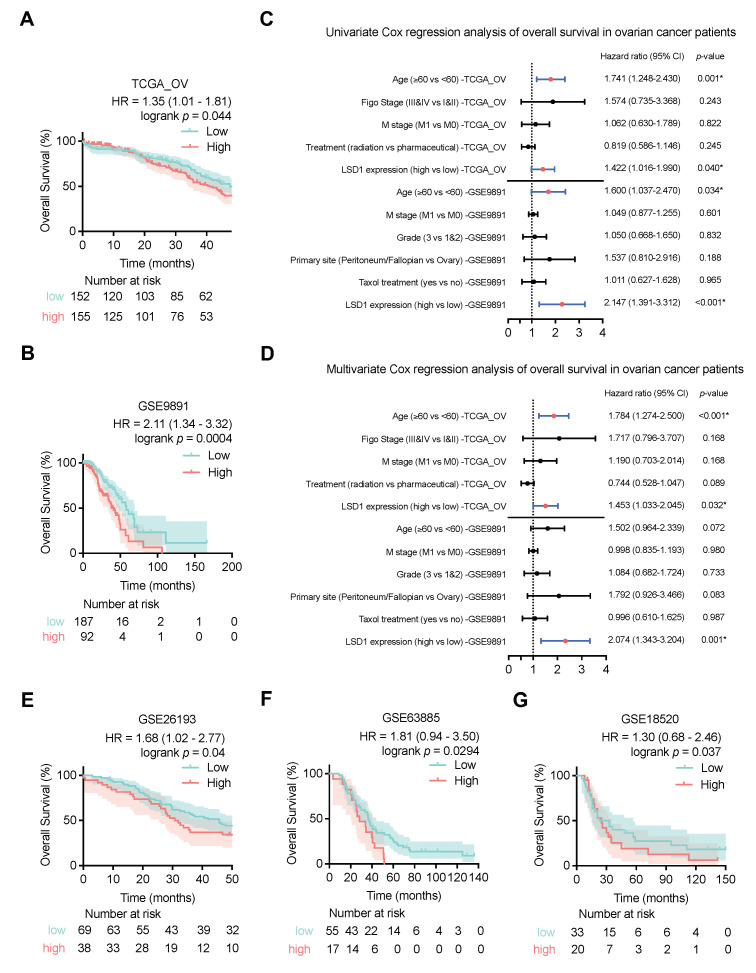
LSD1 is highly related to the prognosis of ovarian cancer patients. (**A**,**B**) KM curves comparing overall survival of ovarian cancer patients of low and high *LSD1* mRNA levels in the cohorts of TCGA_OV (**A**) and GSE9891 (**B**). (**C**,**D**) Forest plots of Cox regression analyses of TCGA_OV and GSE9891 datasets show univariate (**C**) and multivariate (**D**) analyses to determine risk factors associated with overall survival of patients with ovarian cancers. * represents *p* < 0.05. (**E**−**G**) KM curves comparing overall survival of ovarian cancer patients of low and high *LSD1* mRNA levels in the cohorts of GSE26193 (**E**), GSE63885 (**F**), and GSE18520 (**G**).

To assess the correlations between *LSD1* mRNA expression and survival outcomes in ovarian cancer patients, KM curves were generated using clinical survival data of OS from TCGA and GSE datasets. As shown in Figure 3A, high LSD1 expression was more strongly associated with shorter OS in patients with ovarian cancer (*p* = 0.044). Using GSE datasets for validation, the increased LSD1 expression group was found to possess remarkably inferior OS compared with the low LSD1 expression group in GSE9891 (*p* = 0.0004; Figure 3B), GSE26193 (*p* = 0.040; Figure 3E), GSE63885 (*p* = 0.0294; Figure 3F), and GSE18520 (*p* = 0.037; Figure 3G) datasets.

Moreover, to verify the robust prognostic value of LSD1 in terms of OS, univariate and multivariate analyses based on the Cox regression model were performed. Regarding OS, the elevated LSD1 expression was found to be linked with unfavorable OS in patients from the TCGA_OV via univariate analysis (*p* = 0.040, Figure 3C). Multivariate analysis confirmed that high LSD1 expression could independently predict poor OS (*p* = 0.032; Figure 3D). Subsequently, the same analysis further verified that high LSD1 expression was an independent prognostic indicator, performed on GSE9891 (*p* = 0.001; Figure 3D), GSE26193 (*p* = 0.019; Supplementary Figure S2B), and GSE63885 (*p* = 0.034; Supplementary Figure S2B) from the GEO database.

### 3.4. LSD1 Demethylates LC3B and Decreases LC3B Stability

**Figure 4:**
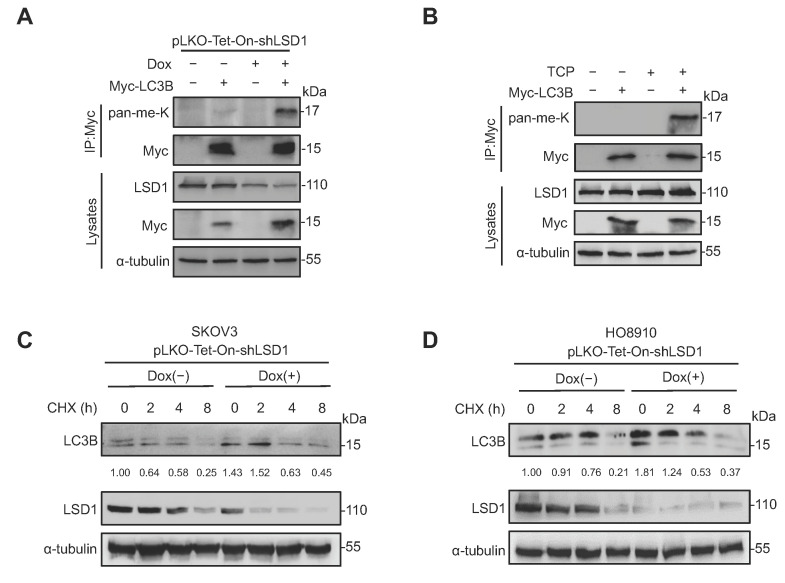
LC3B is demethylated by LSD1, and destabilized by LSD1 expression. (**A**) Dox-induced knockdown of *LSD1* in HO8910 cells was generated. Then, the samples expressing Myc-LC3B protein or not were precipitated using an anti-Myc antibody and analyzed using an anti-pan-me-K antibody to detect methylation of LC3B. (**B**) Inhibition of LSD1 demethylation activity in HO8910 cells was achieved by treatment with TCP. Then the samples expressing Myc-LC3B protein or not were precipitated using an anti-Myc antibody and analyzed using an anti-pan-me-K antibody to detect methylation of LC3B. (**C**,**D**) Western blotting analysis of changes in total LC3B expression in SKOV3 (C) or HO8910 (D) treated (+) or untreated (−) with Dox to induce *LSD1* knockdown with CHX treated for indicated times. The band intensity of total LC3B (LC3B-I and LC3B-II) was quantified, and the corresponding values were labeled below the LC3B Western blot panel. Original Western blot images are available in Supplementary Materials.

To demonstrate whether LSD1 demethylates LC3B, it was shown that the methylation level of LC3B increased in Dox-induced *LSD1*-knockdown HO8910 cells (Figure 4A). Moreover, upon treatment with the LSD1 inhibitor TCP, LC3B methylation increased in HO8910 cells (Figure 4B). This demethylase activity against LC3B suggests that LSD1 may regulate LC3B activity by directly modulating the lysine methylation level of LC3B.

To elucidate the regulation of LC3B mediated by LSD1, the protein levels of LC3B were determined at different time points following treatment with CHX, an inhibitor of protein synthesis in eukaryotes. We found that the Dox-induced knockdown of *LSD1* in SKOV3 and HO8910 cells suppressed the degradation of total endogenous LC3B proteins, where the total amount is represented by the sum of LC3B-I (upper band) and LC3B-II (lower band) (Figure 4C,D). This suggests that LSD1 decreases LC3B stability by accelerating its degradation. The discrepancy in LC3B-I and LC3B-II levels between Figure 4C,D is likely due to inherent differences in basal autophagy activity and the regulatory effects of *LSD1* knockdown on autophagic flux in SKOV3 and HO8910 cell lines, influenced by their distinct genetic backgrounds.

### 3.5. LSD1 Is Inversely Correlated with LC3B at the Protein Level in Clinical Ovarian Cancer Samples

**Figure 5:**
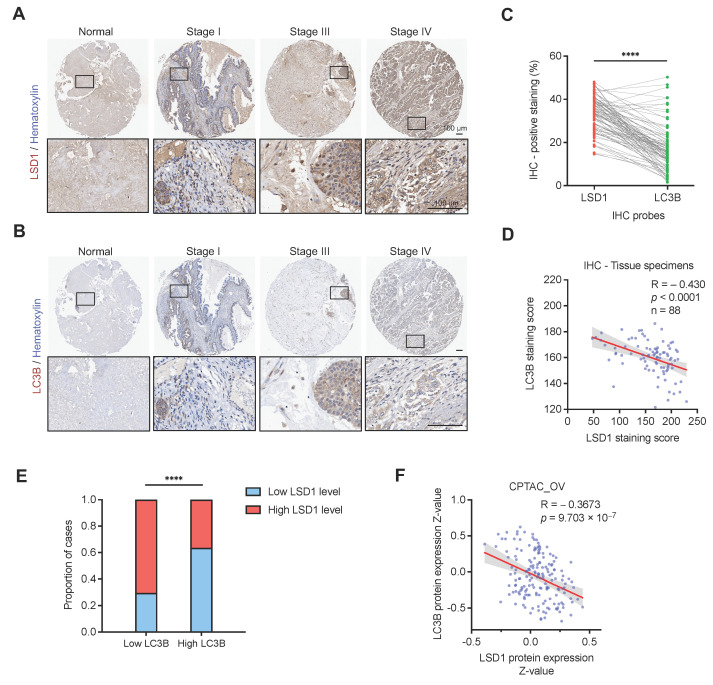
LSD1 expression negatively correlates with LC3B protein level in ovarian cancer. (**A**,**B**) Representative IHC images of LSD1 (**A**) and LC3B (**B**) staining in different stages of the same cohort of ovarian cancer and normal ovary tissues. Scale bar, 100 μm. The rectangle boxes shown in each IHC image in the upper panel indicate the areas that have been magnified in the lower panel for clearer visualization of the antibody staining. (**C**) Paired comparison of IHC-positive staining percentages between LSD1 and LC3B in the same cohort of ovarian tumor tissues. A paired *t*-test (Wilcoxon signed-rank test) was used for *p*-value calculation. (**D**) Pearson correlation between LSD1 and LC3B staining scores in ovarian tumor tissues. R, Pearson correlation coefficient; center line, mean of best-fit line; the shadow indicates 95% confidence interval. (**E**) high LSD1 level significantly correlates with low LC3B level. (**F**) A Pearson correlation coefficient was calculated to determine the correlation between LSD1 and LC3B in the CPTAC protein data of ovarian cancer patients. **** represents *p* < 0.0001.

Given that LSD1 demethylates LC3B, leading to its further degradation, we further sought to determine the clinical correlation between LSD1 and LC3B. To achieve this, we examined IHC staining of LSD1 and LC3B in the same cohort samples of ovarian tumor and normal ovary tissues (Figure 5A,B). The paired comparison of IHC-positive staining percentages between LSD1 and LC3B in the same cohort of ovarian cancer patients revealed that LSD1 had a significantly higher percentage of positive staining compared to LC3B in 83 out of 88 cases (Figure 5C). This indicates a statistically significant association between increased LSD1-positive staining and decreased LC3B-positive staining in this patient cohort. Notably, we discovered that LSD1 was inversely correlated with LC3B at protein levels by analyzing the staining scores of IHC samples. (Figure 5D,E). A similar result was also found in the CPTAC database of ovarian cancer (Figure 5F), providing further support that LC3B was post-translationally regulated by LSD1.

## 4. Discussion

In this study, we presented novel findings elucidating the demethylation of the LC3B protein by LSD1, representing the first reported instance of such a modification. Our investigation reveals a direct interaction between LSD1 and LC3B, leading to the demethylation of LC3B by LSD1. Subsequent demethylation events result in the destabilization of LC3B. KM plotter analysis reveals that elevated LSD1 expression is associated with reduced OS duration in ovarian cancer patients. Notably, our data highlight a negative correlation between LSD1 expression and LC3B protein levels in ovarian cancer cases.

The central protein LC3 in autophagy, involved in the progression of a number of human malignancies, could be used as a prognostic marker in cancers such as breast, liver, lung, colorectal [38], and esophageal cancer [39]. We specifically investigated LC3B, a key marker for autophagy and the most frequently studied LC3 subfamily member in cancer research. Prior studies have examined the prognostic significance of different LC3 subfamily members in ovarian cancer. Elevated LC3A levels have been associated with poor prognosis, particularly in clear cell ovarian carcinomas, suggesting subtype-specific relevance [40,41]. Conversely, one study reported that reduced LC3B levels are linked to adverse outcomes in serous carcinoma, consistent with our findings regarding LC3B [42]. Additionally, another study did not identify a significant correlation between LC3 expression and poor prognosis in ovarian cancer [43], highlighting the heterogeneous nature of LC3’s prognostic value. This variability underscores the necessity of considering both LC3 subtype and ovarian cancer subtype in prognostic evaluations.

As with other major cellular pathways, ATG proteins are subject to regulatory PTMs. Phosphorylation is so far the most intensively studied PTM in the autophagy process, followed by ubiquitination and acetylation [15]. An interesting and new area is also now emerging, which appears to complement these more traditional mechanisms, and includes methylation. Recent studies have shown that protein methylation is associated with effects on autophagosome formation, ATG protein expression, and signaling pathway activation, but the details are still unclear [17]. Identification of the full spectrum of methylation and demethylation of ATG proteins, and determination of their impact on autophagy, will be crucial for a better understanding of autophagy regulation, its deficits in diseases such as various types of cancers, and how to exploit this process for therapies. Further investigation of methylase and demethylase-induced regulatory molecular mechanisms of methylation levels of LC3 or other ATG proteins must be performed for serving as a research basis. Based on this research, we can explore how affected autophagy effects lead to the alteration of essential characteristics of tumor cells.

LSD1 is often found at elevated levels in various cancers, where it significantly influences tumor development and progression through its regulatory functions. In prostate cancer, high LSD1 expression promotes tumor growth by demethylating H3K9 and enhancing the expression of oncogenes that drive cell proliferation and survival [44,45]. Elevated LSD1 levels in breast cancer are associated with increased tumor aggressiveness due to its role in regulating epithelial-to-mesenchymal transition and modulating key transcription factors involved in metastasis [46,47]. Similarly, in ovarian cancer, high LSD1 expression correlates with enhanced tumor cell migration by altering the expression of genes associated with invasive behavior [32,35,48]. Inhibiting LSD1 with targeted drugs has emerged as a promising strategy in cancer therapy, as it can reverse aberrant gene expression and enhance the efficacy of other treatments by modulating epigenetic regulation in various malignancies [49,50]. In our study, we observed an upregulation of LSD1 in clinical samples from ovarian cancer patients.

Several limitations in our study must be acknowledged. First, the specific site on LC3B that is demethylated by LSD1 has not yet been identified. Understanding the exact demethylation site is crucial for elucidating the full scope of this regulatory modification. Additionally, the methylase responsible for methylating LC3B at the same site targeted by LSD1 remains unidentified. Identifying this methylase is essential for a comprehensive understanding of the reversible modification mechanism. Furthermore, while we observed a negative correlation between LSD1 expression and LC3B protein levels, the exact degradation pathway of LC3B following demethylation remains unclear. Determining the degradation mechanism is critical to explaining how LC3B levels are regulated in ovarian cancer cells. Further research is also required to explore how LSD1-mediated LC3B reduction and the related autophagy activity influence tumor cell survival, metastasis, and overall behavior. In brief, addressing these gaps will provide a more comprehensive understanding of autophagy’s role in ovarian cancer and may guide the development of novel therapeutic strategies.

## 5. Conclusions

In summary, LSD1 is highly expressed in ovarian tumors and strongly associated with poor survival outcomes in patients. By demethylating LC3B, LSD1 promotes its degradation, resulting in lower LC3B levels, which highlights a negative regulatory relationship between LSD1 and LC3B.

## Data Availability

The original contributions presented in the study are included in the article/Supplementary Material. The data supporting this study’s findings are available on request from the corresponding author.

## Supplementary Materials

The following supporting information can be downloaded at: https://www.mdpi.com/article/10.3390/biom14111377/s1, Figure S1: LSD1 expresses aggressively in ovarian tumors; Figure S2: LSD1 is highly related to survival rate of ovarian cancer patients; Table S1: summary of the LSD1-positive and LSD1-negative ovarian normal and tumor tissue samples in the tissue microarrays; Table S2: association of LSD1 expression with pathological categories of ovarian tumors.
