# Supplementary material for: LSD1 Demethylates and Destabilizes Autophagy Protein LC3B in Ovarian Cancer
Source: Biomolecules. 2024 Oct 29;14(11):1377. doi: 10.3390/biom14111377 (PMC11591952; doi:10.3390/biom14111377)
Supplement: Supplementary file 1 [file biomolecules-14-01377-s001.zip › Supplementary Figures-biomolecules.pdf]

Supplementary Figures

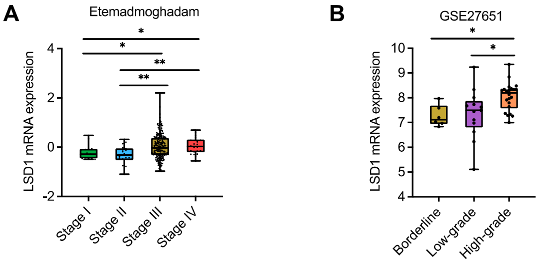

Figure S1. LSD1 ex[illegible]
or grades of ovari[illegible]

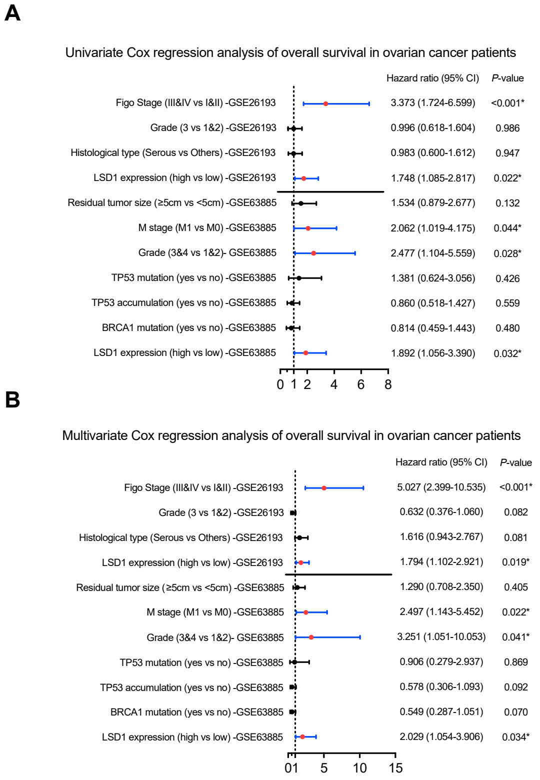

Figure S2. LSD1 is highly related to survival rate of ovarian cancer patients. (A, B) Forest plots of Cox regression analysis of GSE26193 and GSE63885 datasets show univariate (A) and multivariate (B) analyses to determine risk factors associated with overall survival of patients with ovarian cancers.
