# Supplementary material for: LSD1 Demethylates and Destabilizes Autophagy Protein LC3B in Ovarian Cancer
Source: Biomolecules. 2024 Oct 29;14(11):1377. doi: 10.3390/biom14111377 (PMC11591952; doi:10.3390/biom14111377)
Supplement: Supplementary file 1 [file biomolecules-14-01377-s001.zip › Supplementary Tables-biomolecules.pdf]

Supplementary Tables

Table S1. Summary of the LSD1-positive and LSD1-negative ovarian normal and tumor tissue samples in the tissue microarrays.

Tissue	Total	LSD1		*P*
		Positive	Negative	
**Tumor**	88	75(85.2%)	13(14.8%)	0.039*
**Normal**	8	3(37.5%)	5(62.5%)	

Table S2. Association of LSD1 expression with pathological categories of ovarian tumors.

Clinicopathological category	Total Cases	Percentage (%)	LSD1 positive Cases	LSD1 positive %	*P*
**Age**					
≤50	24	27.3	20	83.3	0.046*
＞50	64	72.7	55	85.9	
**Tumor size (cm)**					
≤6	26	29.5	23	88.5	0.404
＞6	62	70.5	52	83.9	
**T category**					
T1	50	56.8	40	80.0	0.095
T2/T3	38	43.2	35	92.1	
**N category**					
N0	39	84.8	29	74.4	0.477
N1	7	15.2	7	100.0	
**M category**					
M0	63	71.6	51	81.0	0.024*
M1	25	28.4	24	96.0	
**FIGO stage**					
I/II	47	53.4	37	78.8	0.040*
III/IV	41	46.6	38	92.7	
**Histologic type**					
Serous	49	55.7	43	87.8	0.573
Mucinous	10	11.4	7	70.0	
Clear cell	9	10.2	8	88.9	
Endometroid	14	15.9	12	85.7	
Metastatic	6	6.8	5	83.3	
