# Supplementary figures and images for: LSD1 Demethylates and Destabilizes Autophagy Protein LC3B in Ovarian Cancer
Source: Biomolecules. 2024 Oct 29;14(11):1377. doi: 10.3390/biom14111377 (PMC11591952; doi:10.3390/biom14111377)

Original WB figures

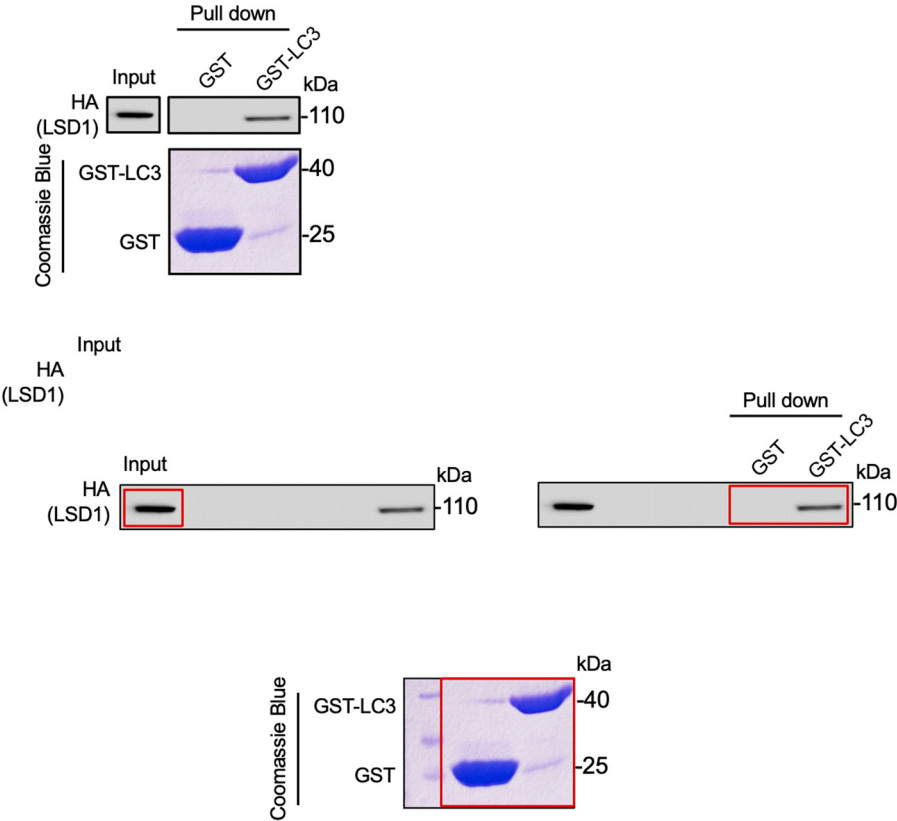

Figure 1A

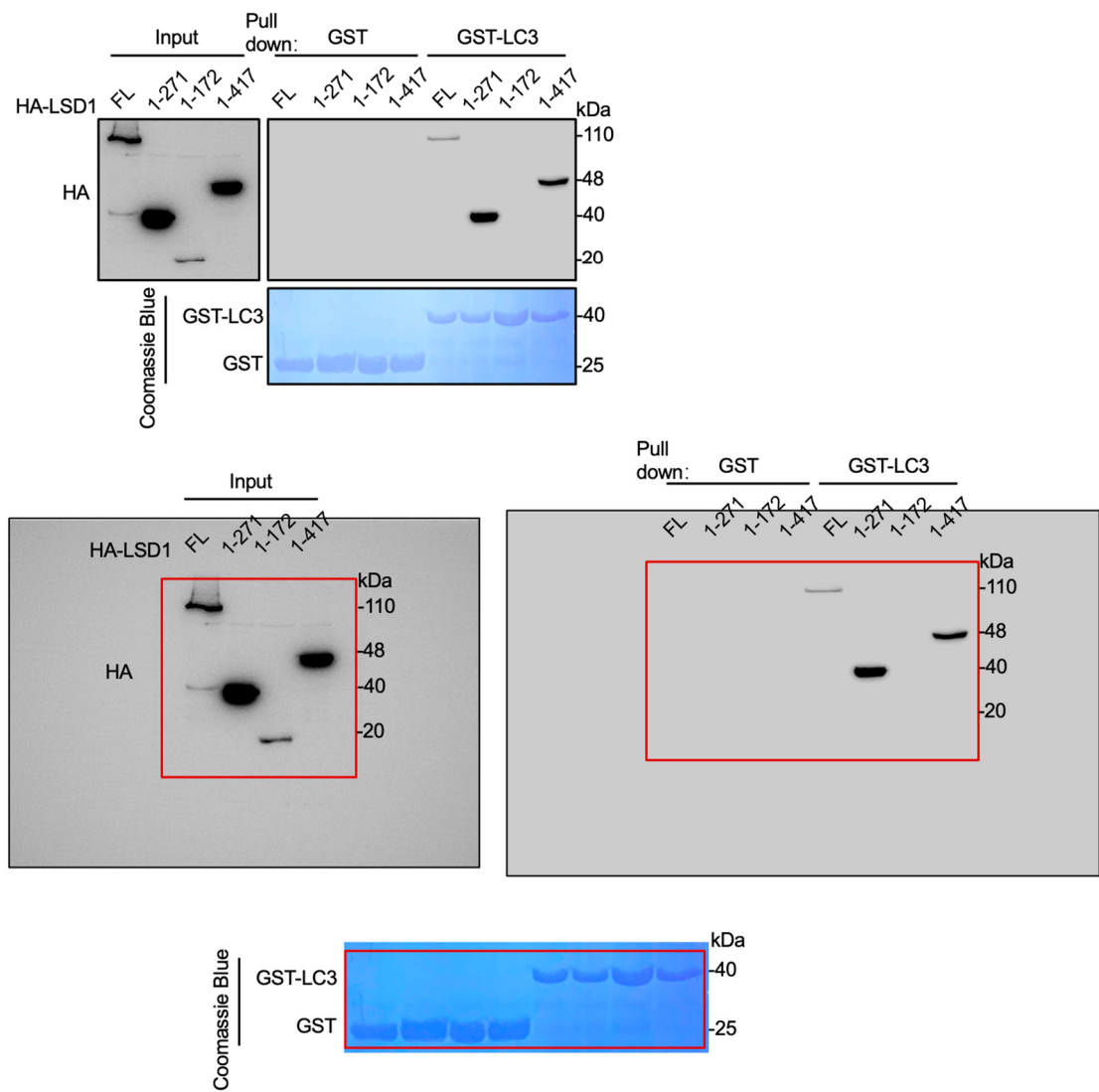

Figure 1D

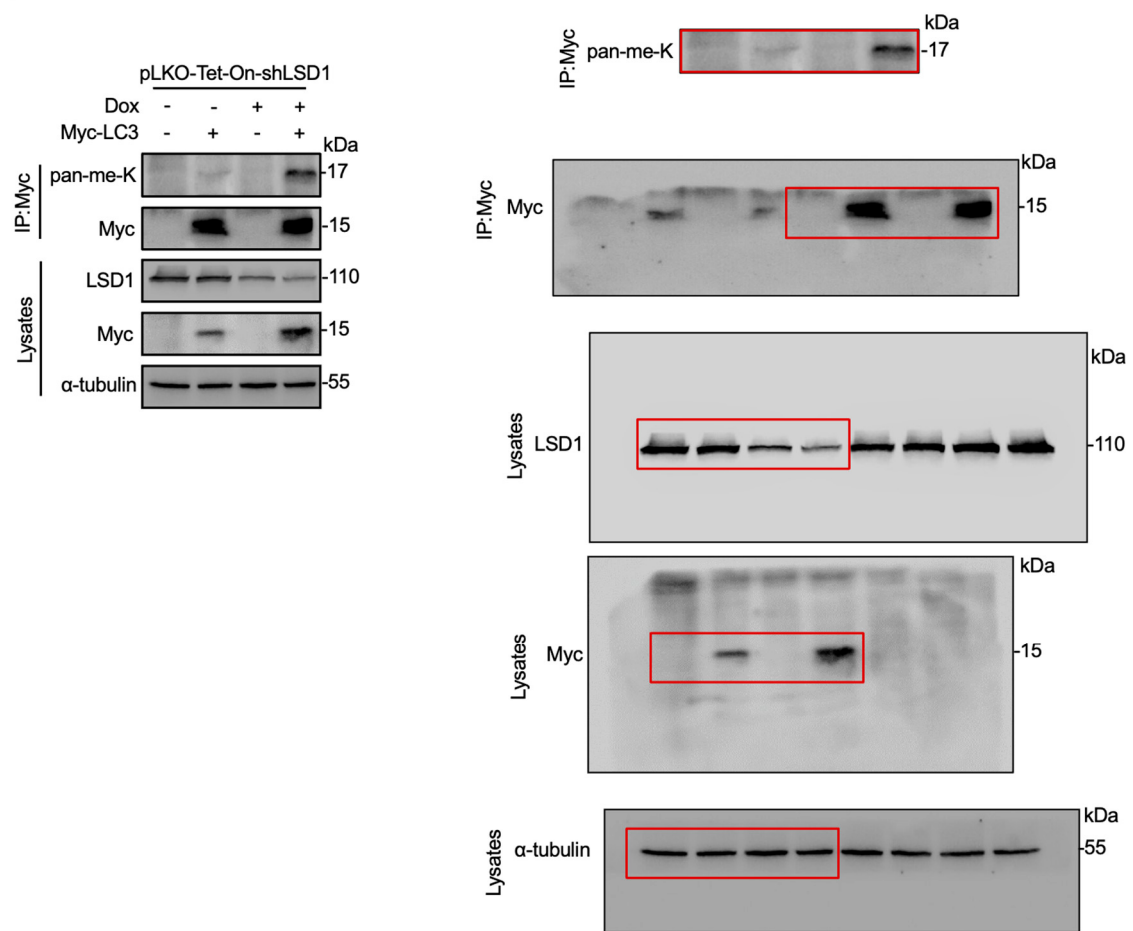

Figure 4A

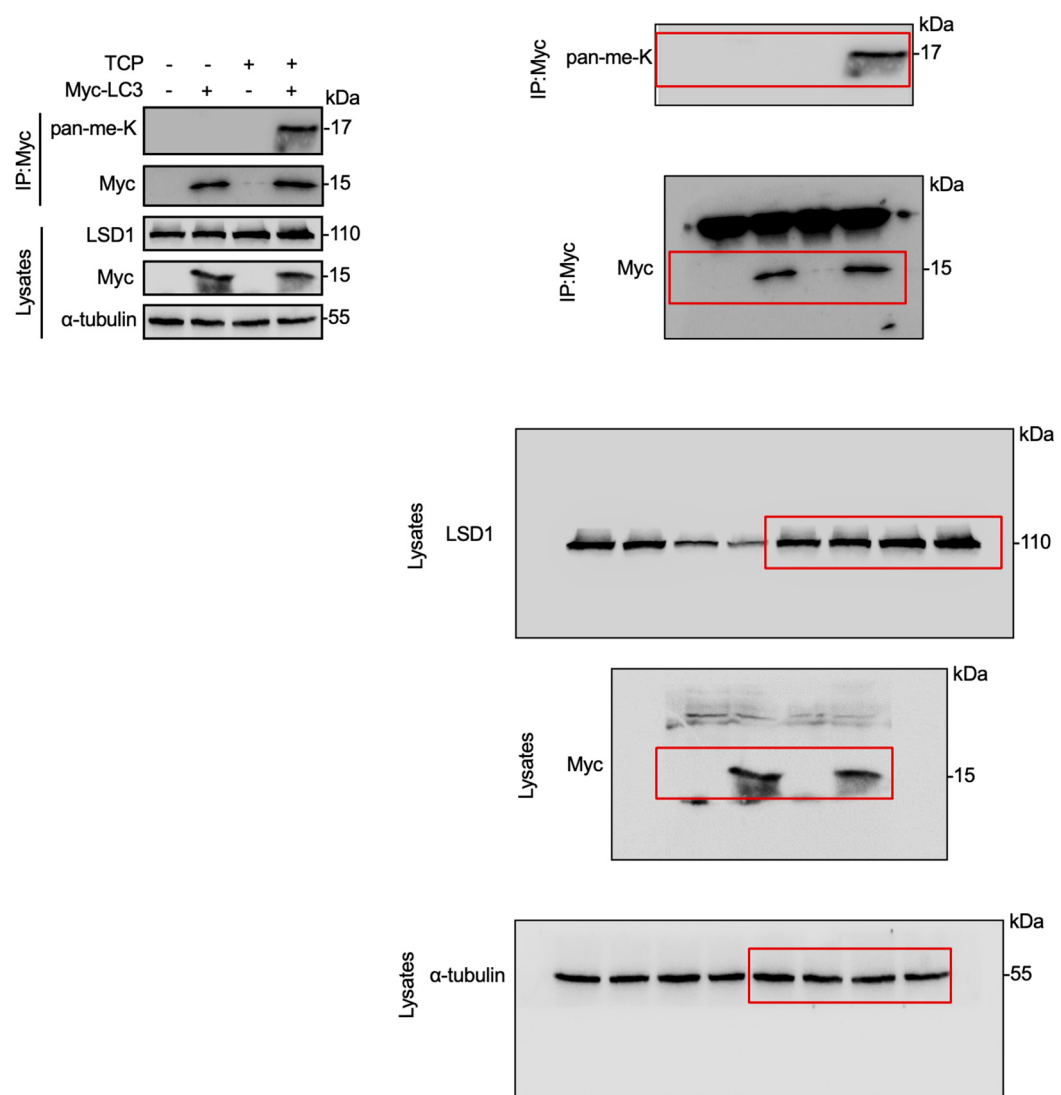

Figure 4B

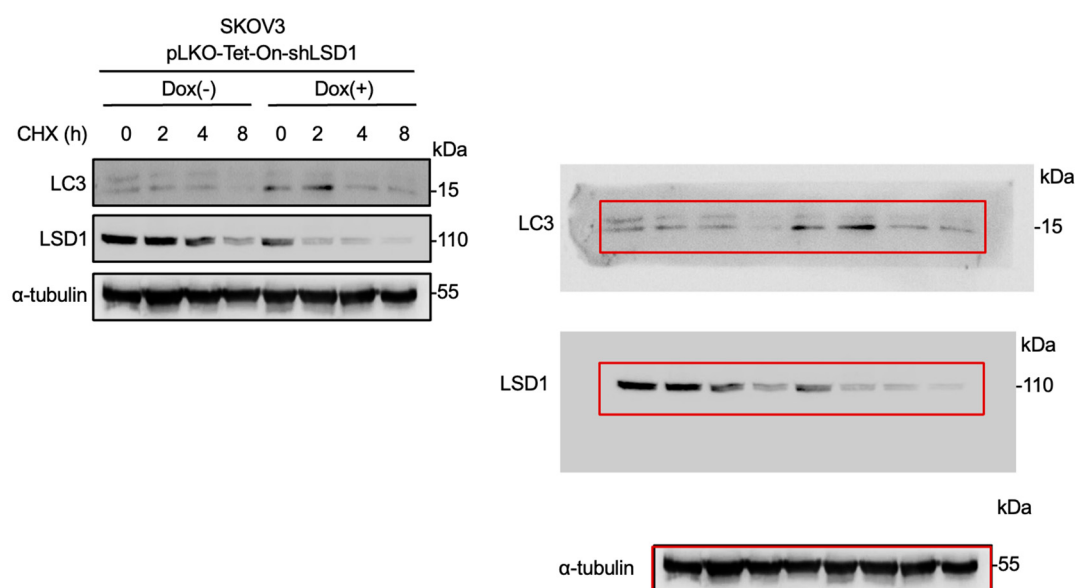

Figure 4C

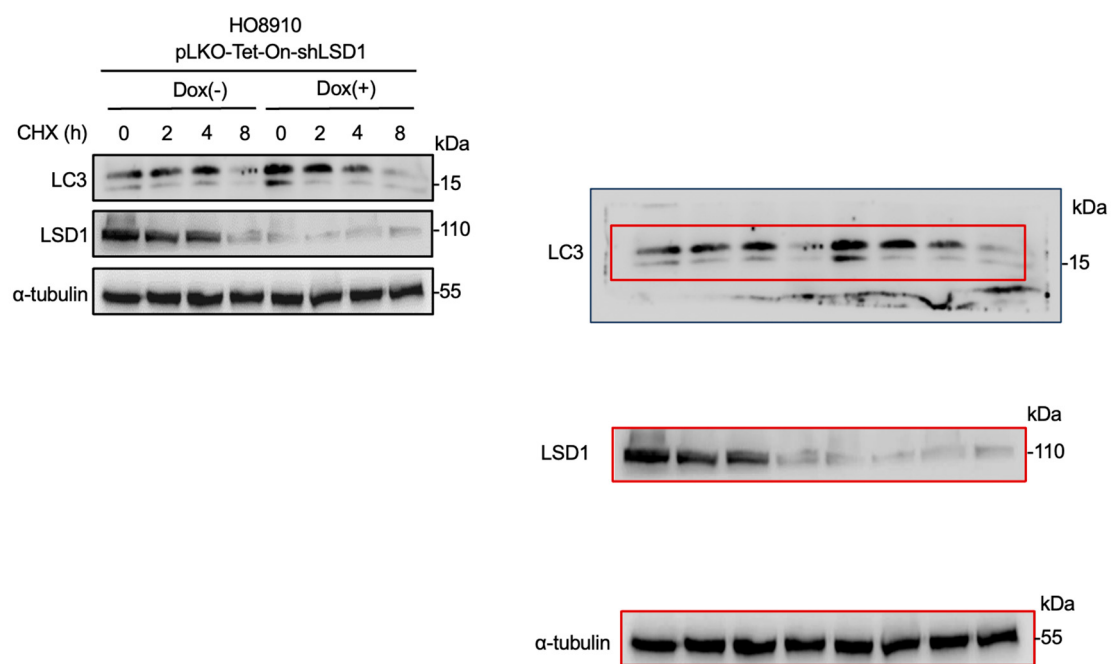

Figure 4D

Supplement: Supplementary file 1 [file biomolecules-14-01377-s001.zip › biomolecules-3150198- original WB figure-highlight.pdf]
