# Supplementary material for: LSD1 Demethylates and Destabilizes Autophagy Protein LC3B in Ovarian Cancer
Source: Biomolecules. 2024 Oct 29;14(11):1377. doi: 10.3390/biom14111377 (PMC11591952; doi:10.3390/biom14111377)
Supplement: Supplementary file 1 [file Zhuoli Biotechnology Company (Shanghai, China) Ethics Committee Review Statement-Original Copy.pdf › Zhuoli Biotechnology Company (Shanghai, China) Ethics Committee Review Statement-Original Copy.pdf]

SHLLS-BA-22101102

	上海卓立生物科技有限公司<br>Shanghai Zhuoli Biotech Company	Barcode	
	伦理委员会审核意见书	编号/Ctl No.	ZLL-15-01
		版本/Version	2.0

项目名称	组织芯片产品开发及应用		
项目来源	上海卓立生物科技有限公司	项目承担单位	上海卓立生物科技有限公司
项目编号	HPan-tumor-TMA	项目负责人	赵传

审核结果

参加伦理委员会会议 __7__ 人，投票结果：

A） 同意 __7__ 票

B)修正后再审查 __0__ 票

C)不同意 __0__ 票

D)暂停或终止 __0__ 票

伦理委员会审查意见：

可参考以下伦理审查内容填写

该项目的知情同意书是否符合规范，知情同意程序是否合理.

该项目的实施是否增加了患者的临床风险.

该项目所产生的样本是否仅被用于科研项目，或经相关主管部门审核通过的国际国内相关产品研发合作项目（如新药研发等），而非不合规范的商业用途.

病人的隐私权在项目设计中得到了充分的保护.

记录人签名： [illegible]<br><br>2022年 10月 11 日	主任委员签名：<br>（伦理委员会盖章）<br>上海卓立生物科技有限公司 伦理委员会<br><br>2022年 10月 11 日
